# The Hospital World

**Published:** 1910-05-14

**Authors:** 


### The Hospital World.

### Elections and Appointments.

Mr. O. F. Read has been reappointed the Hon. Treasurer and Mr. G. L. Betts the Hon. Secretary of the Mildenhall Cottage Hospital.

Mr. Willis Bund has been re-elected president, Mr. Cherry hon. treasurer, and Mr. Holder secretary of the Worcestershire Consumption Hospital.

Dr. R. Sevestre and Mr. Cecil Marriott, F.R.C.S., have been appointed by the honorary medical staff of the Leicester Infirmary as its representatives on the House Committee.

The Earl of Dartmouth has been re-elected President of the Miller General Hospital, Greenwich Road, and Mr. Hamilton Benn, M.P., has been re-appointed Treasurer.

After some discussion Mr. W. Allard, Chairman of the Sanitary Committee of the Surrey County Council, has been appointed Chairman of the Joint Committee of the Surrey Small-pox Hospital.

Mr. E. Entwhistle, chairman, and Mr. J. T. Travis Clegg and Mr. John Moon, the retiring members of the Board of Management of St. John's Hospital of Manchester and Salford for the Ear and Eye, have been re-elected.

The Bristol Eye Dispensary Committee, consisting of Mr. C. E. Barry, Mr. A. W. Blake, Mr. Edward Harvey, jun., Mr. Meyrick W. Heath, Miss Barton Johnson, Mr. W. Moline, Dr. A. Ogilvy, Mr. A. W. Prichard, Mr. W. W. Tothill, and Mr. P. J. Worsley, have been re-elected at the recent annual meeting.

The Lord Mayor of Birmingham (Mr. Alderman Bowater) has been elected president of the Birmingham Hospital Sunday Fund, and the Archdeacon of Aston, the Rev. W. S. Houghton, the honorary clerical secretaries, Mr. Frank Impey, the honorary lay secretary, and Mr. Howard Lloyd, honorary treasurer, have been reappointed.

Mr. W. D. James has been re-elected a member of the committee of the Governors of the West Sussex, East Hants, and Chichester Infirmary. Major Jellicorse was chosen to fill a vacancy, and the following seven Governors have been added to the committee: Prebendary J. Fraser, the Rev. J. C. B. Fletcher, Major Hankey, Captain Grant, Mr. E. H. Lewis, Mr. A. J. Marshall (formerly Secretary), and Lady Gifford.

Lord Henry Bentinck has been elected president and Mr. Bertram A. Smith re-elected treasurer of the Nottingham General Hospital. Mr. T. L. K. Edge is to fill the vacant trusteeship, and Mr. James Forman, Mr. T. A. Hill, the Rev. R. Holden, Mr. G. Richards, Mr. B. Stiebel, Mr. C. W. Birkin, and Mr. Jno. Terry are to act on the monthly board. The special board for the election of honorary medical officers has been appointed as follows: To represent the Hospital Sunday movement: the Rev. Canon Baigent, the Rev. J. C. Grant, Dr. Hill, Mr. J. M. Lloyd Thomas, and Mr. J. Wright. To represent the Hospital Saturday movement: Mr. W. Broughton, Mr. R. B. Hallam, Mr. J. T. Cook, Mr. J. Smedley, and Mr. S. F. Weston. To represent the general body of governors: Sir W. A. Blain, Sir Jesse Boot, Major-General Warrand, Mr. W. J. Douse, Mr. R. FitzHugh, Mr. W. Foster, Mr. G. Goodall, Mr. J. A. H. Green, Mr. R. Halford, Mr. H. G. Ley, Mr. J. Player, Mr. H. E. Thornton, Mr. C. H. Torr, Mr. J. Walker, and Mr. M. Weinberg.

### Personalia.

The death of the Rev. W. H. C. Palmer, who had been a member of the Committee of the Coventry and Warwickshire Hospital, was regretfully alluded to at the April meeting, and the secretary, Mr. J. A. Rudd, has forwarded to Mrs. Palmer and her family an expression of the sympathy of the Committee. The vacancy on the General Committee has been filled by Admiral Bacon.

Dr. Donald J. Mackintosh, M.V.O., has been nominated by the British Medical Association to visit and to report upon the condition and administration of the Bolton Infirmary. This appointment has been made in accordance with the request of the Infirmary Board, referred to in The Hospital of March 12, page 700. Dr. Mackintosh, the well-known superintendent of the Western Infirmary, Glasgow, is the author of "Construction, Equipment, and Management of a General Hospital," on which we published a special article on November 27, 1909.

Captain W. Carey, R.N., who has been for some years Hon. Secretary of the Royal Hants County Hospital, has now sent in his resignation. Captain Carey's secretaryship has been marked by many quiet but invaluable reforms in the economic working of the hospital, which deserve gratitude from a public that ought not to need coaching in expressing it. Captain Carey and the hospital would have heard quickly enough of any extravagance on their part, and the public acknowledgment of his services by the Governors the other day show that they on their side have appreciated him.

After holding the office of Chairman of the Committee of Governors of the West Sussex, East Hants, and Chichester Infirmary for some eight years, and after having been a member for a still longer period, Chancellor Davey has refused to be re-appointed. In spite of a unanimous vote electing him he held out, saying, "It has been a great pleasure to me to work for the Infirmary, but I agree with Prebendary Fraser in the old rule which precludes a member of the committee from serving again for six months after he has sat on that committee for six consecutive years." Major Chauncey only voiced the entreaty of the meeting when he said that the Chancellor's sympathy and tact with the patients and his sound advice would be a genuine loss to the Committee.

The Bury and District Joint Hospital Board has lost the services of two prominent and hard-working members, and Mr. Chambers, who was in the chair at the recent half-yearly meeting, referred to their record in terms that are worth recollection. Mr. Garnett and Mr. Lomax are the retiring members, the former after twelve years' work, and in speaking of Mr. Garnett the chairman read a letter from an old friend, which said: "The valuable services rendered by him during the period will, I am sure, be readily admitted by all his colleagues; and the respect and good will by which he inspired them all will make his removal felt as a personal loss." Mr. Garnett probably has had more to do with the Board's work than any other man, and the attention he has given to the comfort of patients in the Hospital Board's hospitals has made his name a popular one in the district. It was Mr. Garnett's influence, again, which led to the splendid present of the Holcombe Hall estate from Mr. and Mrs. Aitken. Mr. Lomax's work has been recorded on the minutes of the Board, and both he and Mr. Garnett will have the satisfaction of feeling that their work has been appreciated, as it should be, for such workers form the backbone of the hospital world.

